# Development of Therapeutic Chimeric Uricase by Exon Replacement/Restoration and Site-Directed Mutagenesis

**DOI:** 10.3390/ijms17050764

**Published:** 2016-05-20

**Authors:** Guangrong Xie, Weizhen Yang, Jing Chen, Miaomiao Li, Nan Jiang, Baixue Zhao, Si Chen, Min Wang, Jianhua Chen

**Affiliations:** School of Life Science and Technology, China Pharmaceutical University, No. 24, Tong Jia Xiang, Nanjing 210009, China; 15311030132@stu.cpu.edu.cn (G.X.); 14211030582@stu.cpu.edu.cn (W.Y.); 1020071789@cpu.edu.cn (J.C.); 15211030519@stu.cpu.edu.cn (M.L.); 13211030516@stu.cpu.edu.cn (N.J.); 1244724@stu.cpu.edu.cn (B.Z.); 15211030583@stu.cpu.edu.cn (S.C.)

**Keywords:** uricase, exon replacement/restoration, site-directed mutagenesis

## Abstract

The activity of urate oxidase was lost during hominoid evolution, resulting in high susceptibility to hyperuricemia and gout in humans. In order to develop a more “human-like” uricase for therapeutic use, exon replacement/restoration and site-directed mutagenesis were performed to obtain porcine–human uricase with higher homology to deduced human uricase (dHU) and increased uricolytic activity. In an exon replacement study, substitution of exon 6 in wild porcine uricase (wPU) gene with corresponding exon in *dhu* totally abolished its activity. Substitutions of exon 5, 3, and 1–2 led to 85%, 60%, and 45% loss of activity, respectively. However, replacement of exon 4 and 7–8 did not significantly change the enzyme activity. When exon 5, 6, and 3 in *dhu* were replaced by their counterparts in *wpu*, the resulting chimera H_1-2_P_3_H_4_P_5-6_H_7-8_ was active, but only about 28% of wPU. Multiple sequence alignment and homology modeling predicted that mutations of E24D and E83G in H_1-2_P_3_H_4_P_5-6_H_7-8_ were favorable for further increase of its activity. After site-directed mutagenesis, H_1-2_P_3_H_4_P_5-6_H_7-8_ (E24D & E83G) with increased homology (91.45%) with dHU and higher activity and catalytic efficiency than the FDA-approved porcine–baboon chimera (PBC) was obtained. It showed optimum activity at pH 8.5 and 35 °C and was stable in a pH range of 6.5–11.0 and temperature range of 20–40 °C.

## 1. Introduction

Urate oxidase (uricase; EC 1.7.3.3; Uox) is responsible for the first step of degradation of uric acid into more water-soluble allantoin that can be more readily excreted through the kidneys [1,2,3]. Uox is found in all three domains of life, but not in all five genera of hominoids (humans, chimpanzees, orangutans, gorillas, and gibbons). As a result, uric acid is the end product of purine metabolism in hominoids, resulting in a 10-fold increase of serum uric acid level compared to non-primate mammals [4,5]. Uric acid production and excretion are normally balanced in humans. Hyperuricemia results from an imbalance between the rates of production and excretion of uric acid [6]. Due to the low solubility (6 mg/dL, 20 °C) of uric acid, long-term hyperuricemia leads to destructive crystalline urate deposits around joints, in soft tissues, and in some organs, causing a number of disorders, including gout and urate nephropathy associated with tumor lysis syndrome [7,8]. Studies have also shown that hyperuricemia in humans can increase the risk of cardiovascular diseases, chronic nephropathy, impaired renal function, hypertension, and stroke [9,10].

Due to those severe complications of hyperuricemia, its therapeutic countermeasures have attracted great attention in the medical community. There are currently three major classes of drugs available for reducing uric acid level, namely, uricosuric drugs, uric acid synthesis inhibitors, and recombinant Uox preparations [11,12]. Uricosuric agents promote uric acid excretion, but are ineffective if renal function is impaired. Allopurinol, a strong inhibitor of xanthine oxidase, is the mainstay of therapy in patients with tophaceous gout, renal insufficiency, leukemia, and some inherited disorders. However, some patients are refractory to the therapy and ~2% of patients receiving allopurinol develop allergic reactions, even severe hypersensitivity syndrome (~0.4%) [13,14].

Rasburicase (Fasturtec^®^), a recombinant form of Uox from *Aspergillus flavus*, is the first marketed Uox preparation for the treatment and prophylaxis of acute hyperuricemia resulting from tumor lysis syndrome in children with cancer [15,16]. Its outstanding ability to decrease uric acid level and dissolve tophi makes Uox-based therapy a more promising strategy for the treatment of hyperuricemia [17,18]. However, the clinical use of rasburicase is limited by its immunogenicity and short half-life [19]. In 2010, pegloticase (Krystexxa), a PEGylated chimeric porcine-baboon Uox, was approved by the USA Food and Drug Administration (FDA) for treatment of chronic gout in adult patients refractory to conventional therapy [20,21]. With a higher sequence homology to hypothetic human uricase and PEGylation, the immunogenicity of pegloticase is significantly reduced and half-life prolonged. In addition to the FDA approved porcine–baboon chimera (PBC), investigations on several other chimeric uricases have also been reported, such as canine–human chimeric uricase [22] and porcine–human chimeric uricase [23]. The development of therapeutic uricase for human use is an intractable challenge as activity, stability, and immunoreactivity should all be taken into consideration. As such, the medical community has a strong interest in developing a recombinant “human-like” uricase to treat hyperuricemia and gout [24].

The pseudogene of human uricase was mapped to chromosome 1p22 [25].The eight exons in *dhu* collectively give a nucleotide sequence of 915 bp and code for 304 amino acids. Although this enzyme is lost in hominoid primates, it is crucial in controlling uric acid levels in other mammals. Porcine uricase, with eight exons and a high homology (87.5%) to human uricase, was detected to be the most active among mammalian uricases. In this report, exon replacement was performed on wild porcine uricase (wPU) gene with corresponding exon from *dhu* to investigate the overall detrimental effect of each exon on the enzyme activity. Subsequently, exon restoration was performed on *dhu* by replacing the three most deleterious exons with corresponding exons from *wpu*. After further site-directed mutations at amino acid residues 24 and 83, chimeric uricase H_1-2_P_3_H_4_P_5-6_H_7-8_ (E24D & E83G) with increased activity and higher homology with dHU was obtained.

## 2. Results

### 2.1. Expression and Purification of *dHU* and *wPU*

**Figure 1:**
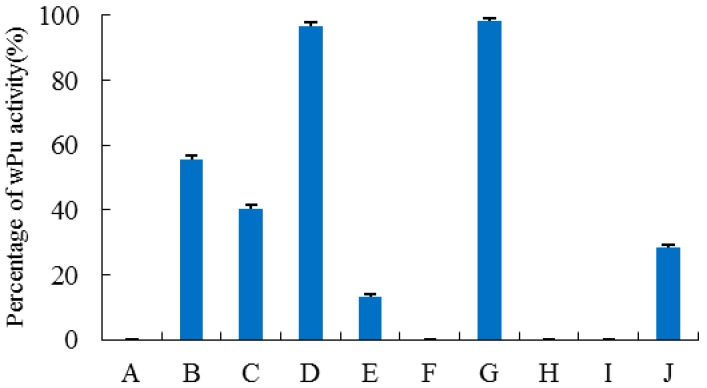
Enzymatic activity of porcine–human chimeras generated during exon replacement and exon restoration study. The enzymatic activity is expressed as a percentage of the activity observed for wPU. (*n* = 5, error bars represent standard deviation). (A) dHU; (B) H_1-2_P_3-8_; (C) P_1-2_H_3_P_4-8_; (D) P_1-3_H_4_P_5-8_; (E) P_1-4_H_5_P_6-8_; (F) P_1-5_H_6_P_7-8_; (G) P_1-6_H_7-8_; (H) H_1-5_P_6_H_7-8_; (I) H_1-4_P_5-6_H_7-8_; (J) H_1-2_P_3_H_4_P_5-6_H_7-8_.

**Figure 2:**
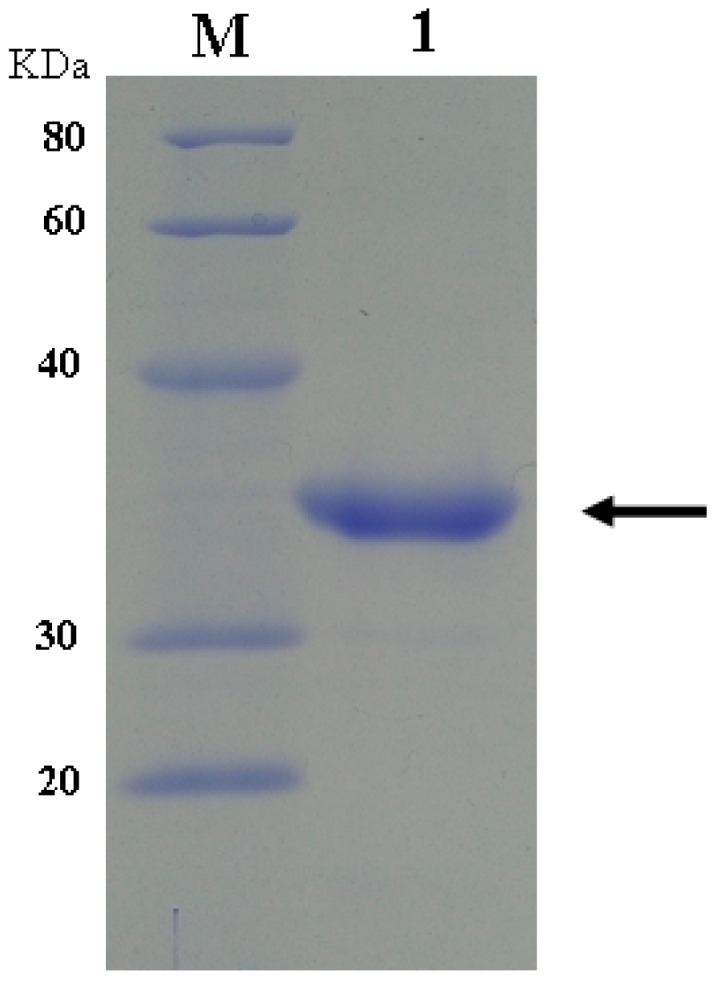
Homogeneity of purified wPU by SDS-PAGE analysis. Lane 1: purified wPU; lane M: molecular weight markers.

Two stop codons in the HU gene were intentionally replaced by the codon of arginine during the chemical synthesis of *dhu*. Both *dhu* and *wpu* were confirmed by DNA sequencing and inserted into pET-22b(+) vector. After transformation into *E. coli* BL 21, positive transformants were screened and cultivated for induced expression of the enzyme. As expected, dHU did not show any catalytic activity in the enzymatic assay (Figure 1). wPU was successfully expressed and purified to homogeneity, as illustrated by the distinct band at 34 kDa on SDS-PAGE in Figure 2.

### 2.2. Construction, Purification, and Enzymatic Assay of Porcine–Human Uricases

Since exon 1 of human uricase is only 30 bp, exons 1 and 2 were investigated collectively, as were exons 7 and 8. By SOE-PCR (splicing by overlap extension-PCR), Exons 1–2, 3, 4, 5, 6, 7–8 of *wpu* were respectively replaced by the corresponding exon in *dhu*. All these chimeric uricases were successfully constructed, expressed, and purified. As shown in Figure 1, replacement of different exons led to varying deleterious effects on enzyme activity. Substitutions of exon 4 and exons 7–8 in *wpu* did not cause significant change in activity. However, when exon 6 of *wpu* was replaced by the corresponding exon of *dhu*, chimera P_1-5_H_6_P_7-8_ totally lost its activity. Replacement of exon 5 in *wpu* by the corresponding exon in *dhu* significantly deteriorated the activity, with only 14.3% activity retained in chimera P_1-4_H_5_P_6-8_. Replacement of exons 1–2 and exon 3 in wPU was also destructive, but the activity of H_1-2_P_3-8_ and P_1-2_H_3_P_4-8_ were moderate compared with that of wPU.

For the resurrection of dHU, a series of exon restoration experiments were carried out. Exons from *wpu* were substituted for the corresponding exons in *dhu* one after another, from the most detrimental exon to the least destructive ones. To our surprise, both H_1-5_P_6_H_7-8_ and H_1-4_P_5-6_H_7-8_ did not show any activity. When the two most detrimental exons in *dhu* were replaced by functional exons from *wpu*, it was still not enough to recover any activity. Only when exon 3 was further replaced did chimera H_1-2_P_3_H_4_P_5-6_H_7-8_ begin to show certain activity, about 28% of wPU.

In the assay of kinetic parameters, the most satisfactory results were observed with H_1-2_P_3_H_4_P_5-6_H_7-8_ (E24D & E83G). As shown in Table 1, H_1-2_P_3_H_4_P_5-6_H_7-8_ (E24D & E83G) displayed a smaller *K_m_* than dHU, wPU, and PBC, indicating enhanced activity, which is more desirable to clinical application. It also showed the highest catalytic efficiency, as indicated by *k_cat_/K_m_*. Based on amino acid sequence alignment, H_1-2_P_3_H_4_P_5-6_H_7-8_ (E24D & E83G) is 91.45% homologous to dHU.

### 2.3. Multiple Sequence Alignment and 3D Structure Modeling

**Figure 3:**
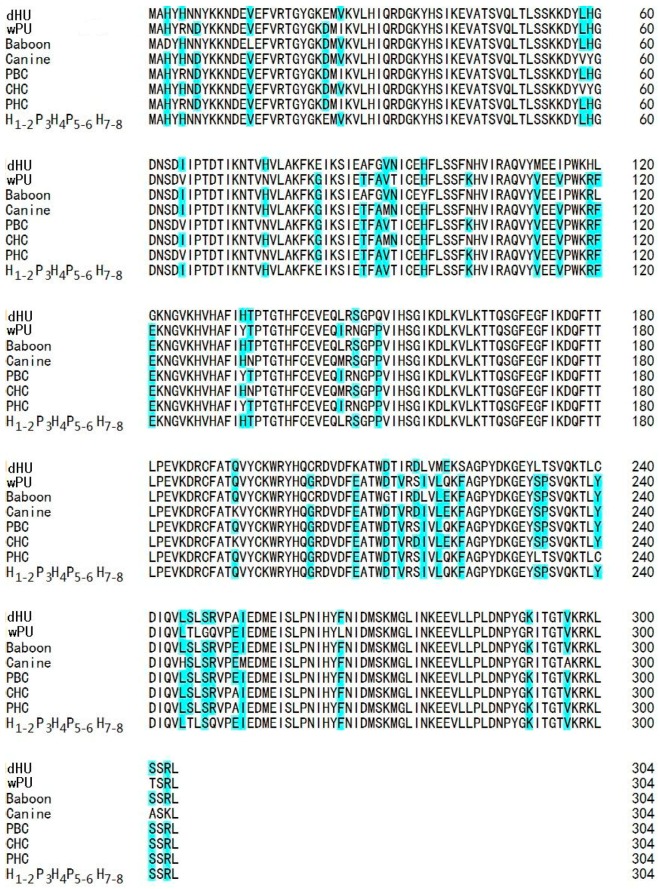
Alignment of deduced protein sequences of H_1-2_P_3_H_4_P_5-6_H_7-8_ with nonfunctional dHU and six functional Uox. Protein sequences are given as standard single-letter designations. DNA sequences of dHU (AB074326.2 with correction of the two stop codons), baboon uricase (BAB91554.1), canine uricase (NP_001069116.1), and porcine uricase (NP_999435.1) are retrieved from GenBank. DNA sequences of pthe orcine–baboon chimera (PBC), porcine–human chimera (PHC), and canine–human chimera (CHC) were obtained from related patents. Inconsistent amino acid residues are highlighted with light blue.

In Figure 3, the amino acid sequence of H_1-2_P_3_H_4_P_5-6_H_7-8_ was aligned with the amino acid sequences of dHU, three mammal uricases (porcine, baboon, and canine), and three chimeric uricases (porcine–human, porcine–baboon, and canine–human). Amino acid residues of inconsistence across these sequences were highlighted. All these inconsistent residues were shared by both functional sequences and nonfunctional sequence except amino acid residues 24 and 83. D24 and G83 are found in all the functional uricases in the alignment, while the two positions in dHU and H_1-2_P_3_H_4_P_5-6_H_7-8_ are E24 and E83, respectively. Therefore, we speculated that the two amino acid residues were the reasons for the low activity observed for H_1-2_P_3_H_4_P_5-6_H_7-8_.

To predict if site-directed mutations at positions 24 and 83 will be favorable for the further restoration of enzyme activity, 3D structures of H_1-2_P_3_H_4_P_5-6_H_7-8_ before and after amino acid replacement at the two positions were constructed and analyzed by homology modeling.

**Figure 4:**
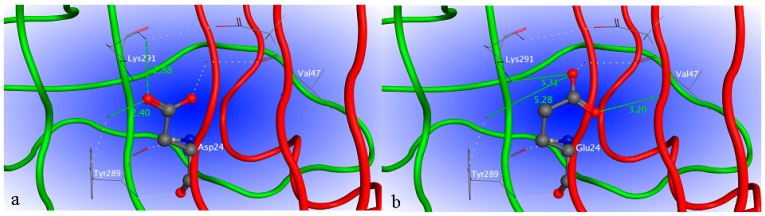
Part of the tertiary structure of H_1-2_P_3_H_4_P_5-6_H_7-8_ before and after amino acid replacement at position 24, constructed using MOE (Molecular Operating Environment). Amino acid residue 24 is shown as a ball and stick structure. The carbon atoms are shown in gray ball, the oxygen atoms are shown in red ball, the nitrogen atoms are in dark blue ball. Backbones of two neighboring monomers are shown in red and green. (**a**) 3D structure with amino acid residue D24; (**b**) 3D structure with amino acid residue E24.

Based on the elucidated crystal structures of uricase from microorganisms and mammals, active uricase folds into a homotetramer (dimer of dimers) in which the monomers are associated by non-covalent binding. Any factor causing the disassociation of the tetramer will lead to exposure of inner hydrophobic amino acid residues and final precipitation of the enzyme, significantly deteriorating the enzymatic activity. As shown in Figure 4, amino acid residue 24 is at the interface of two uricase monomers that form a dimer of the homotetramer. Aspartic acid forms hydrogen bonds with both Lys291 and Tyr289 from the neighboring monomer, which is favorable to the stability of the dimer. However, glutamic acid forms hydrogen bond with neighboring Val47 in the same monomer, consequently moving away from Lys291 and Tyr289 in the adjacent monomer. The increased distance is unfavorable to the association of the two monomers and thus the stability of the dimer.

**Figure 5:**
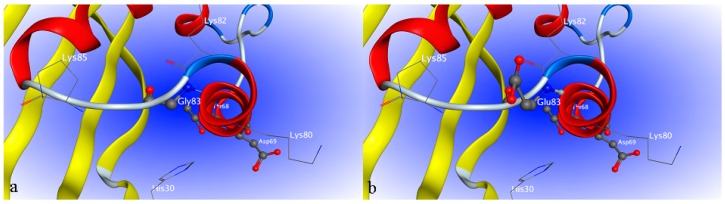
Part of the tertiary structure of H_1-2_P_3_H_4_P_5-6_H_7-8_ before and after amino acid replacement at position 83, constructed using MOE. Amino acid residue 83 is shown as a ball and stick structure. The α-helix is shown in red; the β-sheet is shown in yellow. (**a**) 3D structure with amino acid residue G83; (**b**) 3D structure with amino acid residue E83.

Each monomer of uricase contains four long α-helixes (H1–4) and two short ones (h1–2). As shown in Figure 5, amino acid 83 is located at the connection of two adjacent antiparallel α-helixes H1 and H2. Compared with glycine, glutamic acid increases the steric hindrance at the cornering between the two α-helixes due to its much larger side chain, greatly affecting the spatial orientation of the two helixes. At the distal end of H2, two amino acid residues (Thr68 and Asp69) forming part of the uric acid-binding site are located. Glu83 is very likely to influence the accurate positioning of Thr68 and Asp69 and consequently the enzyme activity.

### 2.4. Site-Directed Mutagenesis

**Figure 6:**
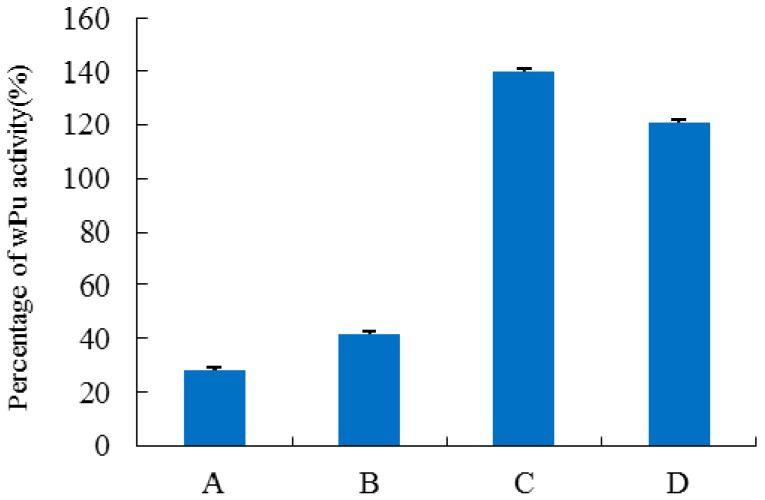
Specific activity determined for mutants obtained by site-directed mutagenesis. Values are expressed as a percentage of the specific activity observed for wPU. (*n* = 5, error bars represent standard deviation). (A) H_1-2_P_3_H_4_P_5-6_H_7-8_; (B) H_1-2_P_3_H_4_P_5-6_H_7-8_ (E24D); (C) H_1-2_P_3_H_4_P_5-6_H_7-8_ (E24D & E83G); (D) PBC.

Based on the predictions by homology modeling, two pairs of primers were designed for the site-directed mutagenesis at positions 24 and 83 in H_1-2_P_3_H_4_P_5-6_H_7-8_ by SOE-PCR. After construction, transformation, and screening by ampicillin, mutants H_1-2_P_3_H_4_P_5-6_H_7-8_ (E24D) and H_1-2_P_3_H_4_P_5-6_H_7-8_ (E24D & E83G) were obtained and purified for activity assay. As shown in Figure 6, when mutation E24D was performed on chimera H_1-2_P_3_H_4_P_5-6_H_7-8_, there was an obvious increase in the activity, although to a level that is still not comparable to that of wPU and PBC. When both mutations E24D and E83G were implemented, the resulting mutant showed significantly increased activity (141% of wPU), which was even higher than that of PBC. During the process of exon restoration and site-directed mutation, the homology of the resulting chimera with dHU was gradually decreased. However, H_1-2_P_3_H_4_P_5-6_H_7-8_ (E24D & E83G) was still 91.45% homologous to dHU, which was higher than both wPU (87.5%) and PBC (89.4%).

### 2.5. Interaction between H_1-2_P_3_H_4_P_5-6_H_7-8_ (E24D & E83G) and Uric Acid

**Figure 7:**
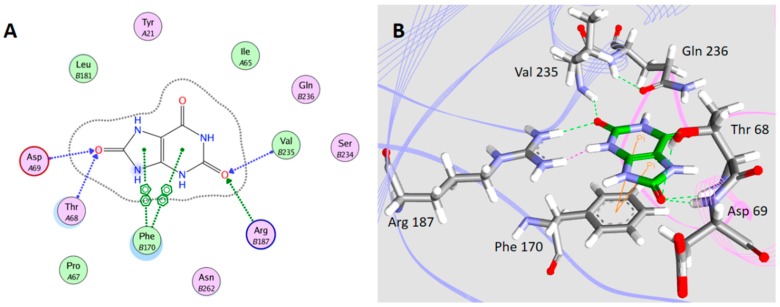
Structure modeling of uric acid to H_1-2_P_3_H_4_P_5-6_H_7-8_ (E24D & E83G) is illustrated in both 2D and 3D view (**A**,**B**). In 2D views, intermolecular interactions between the receptor and the ligand are illustrated, with (**A**) and (**B**) indicating amino acid residues from different subunits. In 3D views, both uric acid and amino acid residues involving in the binding are shown as stick structures. Subunit (**A**) is shown in pink whereas unit (**B**) is shown in blue. The carbon atoms in uric acid are shown in green, the carbon atoms in amino acid residues are shown in gray, the oxygen atoms are shown in red, and the nitrogen atoms are shown in blue.

The interactions between uric acid and H_1-2_P_3_H_4_P_5-6_H_7-8_ (E24D & E83G) were simulated *in silico* (Figure 6). Two types of intermolecular interactions, H-bonding and π–π interaction, were observed between uric acid and the binding pocket of uricase H_1-2_P_3_H_4_P_5-6_H_7-8_ (E24D & E83G). As shown in Figure 7, the H-bonding is formed between T68, D69, R187, V235, and uric acid, while the π–π interaction is formed between F170 and uric acid. This interaction is very similar to that observed for uric acid and uricase of microbial origin, such as *Aspergillus flavus* uricases (PID ID: 4PR8) and *Arthrobacter globiformis* uricase (PID ID: 2YZB). The purine ring of uric acid is tightly held to one monomer of uricase through molecular tweezers built by the side chains of a conserved Arg and a conserved Gln, and is stacked on a conserved Phe. However, more involvement of V235 was observed in the case of our mutant enzyme.

### 2.6. Characterization of H_1-2_P_3_H_4_P_5-6_H_7-8_ (E24D & E83G)

**Figure 8:**
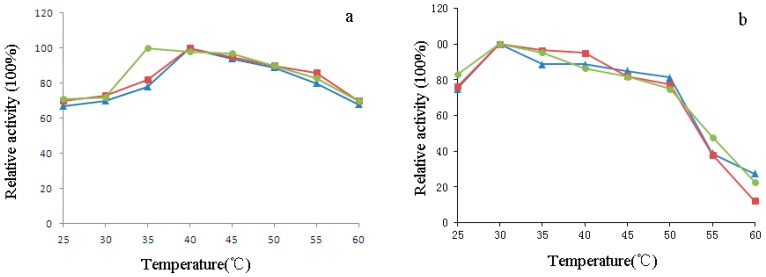
Effect of temperature on uricolytic activity (**a**) and stability (**b**) of wPU (▲), PBC (■), and H_1-2_P_3_H_4_P_5-6_H_7-8_ (E24D & E83G) (●). Enzyme activity at the optimum temperature (activity) or at the beginning of incubation (stability) was assumed to be 100%.

The temperature-activity and -stability profiles of purified H_1-2_P_3_H_4_P_5-6_H_7-8_ (E24D & E83G) are illustrated in Figure 8a,b in comparison with wPU and PBC. H_1-2_P_3_H_4_P_5-6_H_7-8_ (E24D & E83G) was optimally active at 35 °C *vs.* wPU and PBC at 40 °C. After 30 min pre-incubation at different temperatures, all three enzymes showed similar temperature stability profiles. Activity was retained well above 80% in the range from 25–45 °C and substantially lost after pre-incubation at 45–60 °C. However, in the range of 25–35 °C, which is also the temperature range at which clinical application occurs, H_1-2_P_3_H_4_P_5-6_H_7-8_ (E24D & E83G) was slightly more stable than wPU and PBC.

**Figure 9:**
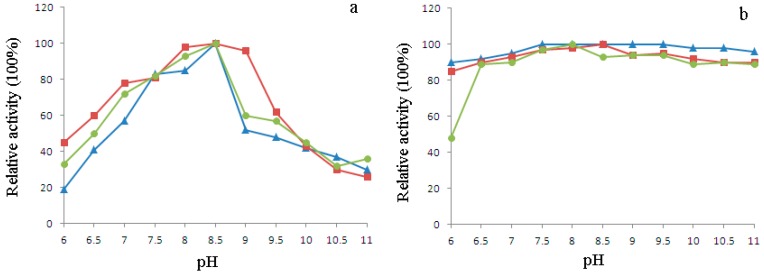
Effect of pH on uricolytic activity (**a**) and stability (**b**) of wPU (▲), PBC (■), and H_1-2_P_3_H_4_P_5-6_H_7-8_ (E24D & E83G) (●). Enzyme activity at the optimum pH (activity) or at the beginning of incubation (stability) was assumed to be 100%.

The optimum pH of H_1-2_P_3_H_4_P_5-6_H_7-8_ (E24D & E83G) was determined over a pH range of 6.0–11.0. According to the pH activity profile shown in Figure 9a, maximum activity was observed for all three uricases at pH 8.5. Pre-incubation in buffer with pH ranging from 6.5 to 11.0 did not cause substantial loss of activity in all three uricases, with more than 80% activity retained. Pre-incubation at pH 6 was deteriorating due to the enzymatic activity of H_1-2_P_3_H_4_P_5-6_H_7-8_ (E24D & E83G), with nearly 50% loss of activity (Figure 9b).

## 3. Discussion

The buildup of serum urate in the blood (hyperuricemia) can have severe consequences for human health. Although Uox preparations are very promising therapies for hyperuricemia and gout, their clinical use has been restricted by immunogenicity, the same as other non-human proteins with useful therapeutic properties. In the development of therapeutic uricase for human use, there should be a balance between activity, stability, and immunoreactivity. Molecular engineering has been frequently applied to construct enzymes with desired properties [26,27,28]. Due to the lack of detailed structure–activity correlation of uricase, rational design is not feasible for engineering the enzyme. Previously, we obtained a more human-like chimeric uricase by DNA shuffling, which was an irrational method of molecular engineering [29]. *dhu* and *wpu* were used as parental genes to generate a diverse chimeric library from which mutant chimeras with desired properties were selected. However, this molecular engineering strategy was relatively tedious and relied greatly on the availability of high-throughput screening methodology.

The activity of human uricase was gradually lost during hominoids’ evolution as a result of the accumulation of both nonsense mutations and missense mutations [30]. It has been proved that human uricase cannot be resurrected simply by replacing the two premature stop codons 33 and 187 present in the pesudogene, leading to the speculation that elimination of other missense mutations are needed to restore the activity. Kratzer *et al.* used site-directed mutagenesis to determine the amino acid substitutions responsible for the decreases in human uricase activity [31]. From the ancestral uricase An19/22 to nonfunctional human uricase, there are 22 amino acids replacement (including codons 33 and 187); nearly all of them are deleterious to enzymatic activity. However, permutation and combination of the remaining 20 missense mutations will result in numerous scenarios, and the construction of these mutants and screening for active ones will become an insuperable task. In this study, we demonstrated successful development of a chimeric uricase with increased homology with dHU and activity through a combination of exon replacement/restoration and site-directed mutagenesis, which could be considered a semi-rational approach.

Silencing or pseudogenization of the human uricase gene is a result of multiple, independent evolutionary events. Although not protein-coding, a pseudogene represents a record of once-functional genetic characteristics. Despite increased sequencing and annotation of pseudogenes, they tend to be ignored. In our study, the sequence of human uricase pseudogene was used for the development of a drug candidate that is more human-like than the FDA-approved PBC, setting a good example for exploring the full potential of pseudogenes. With a high homology with dHU, wPU is also the most active among other mammalian uricases [32]. Both wPU and dHU are composed of 304 amino acids, which are distributed into exons with exactly the same length. Each exon in *wpu* can be considered as the functional counterpart of *dhu*. Therefore, we decided to construct porcine–human chimeric uricase by exon replacement, which allowed for investigation of these mutations collectively within an exon. In the subsequent exon restoration, the activity of dHU was recovered by replacing those significantly deleterious exons. This semi-rational approach circumvents the tedious screening of the irrational approach and allows for the study of exon-based structure–activity correlation.

An exon replacement study showed that exon 6 had the greatest detrimental effect, leading to a total loss of activity. This was consistent with a previous study by Kratzer *et al.* [31]. The three mutations during hominoids’ evolution with the largest deleterious effect on the catalytic activity of uricase are all distributed in exon 6—S232L, Y240C, and F222S. A single mutation of S232L nearly abolished the catalytic activity of uricase An19/22, the ancestral uricase with much higher activity than wPU. The other two mutations prevented uricase solubility and thus the determination of activity [30]. It is very reasonable to conclude that mutations in exon 6 are the predominant reason for the inactivation of HU. From the most deteriorating to the least destructive, exons in *dhu* follow this sequence: exon 6, exon 5, exon 3, exons 1–2, exon 4, and exons 7–8. It is interesting to note that exon 5 in *dhu* was only two amino acids (positions 202 and 208) different from that of wPU; however, a much larger detrimental effect on enzymatic activity was produced than the collective effect of all the eight mutations existing in exon 3. Exons with a significant detrimental effect on human uricase were chosen to be replaced by the corresponding exons in *wpu* to restore dHU activity. After replacement of three exons, moderate activity was detected for chimera H_1-2_P_3_H_4_P_5-6_H_7-8_. However, this activity was too low to have any clinical significance.

In order to further increase the activity of porcine–human uricase to an acceptable level and at the same time retain a high degree of homology to dHU, exon restoration was not continued for exons 1–2, 4, and 7–8. An exon replacement study suggested that mutations in these five exons were generally not so detrimental to the enzyme activity. However, some of them may be more liablethan others. Our strategy was to identify the more liablemutations in these five exons by multiple sequence alignment and homology modeling. Sequence alignment of functional uricase and non-functional uricase revealed that 24E and 83E were shared by nonfunctional dHU and H_1-2_P_3_H_4_P_5-6_H_7-8_ with poor activity, while 24D and 83G were found in all other uricases in the alignment with relatively high activity. We hypothesized that 24D and 83G might be critical for enzyme activity, which was further predicted by molecular modeling. In our analysis of the modeled 3D structures, 24E in H_1-2_P_3_H_4_P_5-6_H_7-8_ results in increased distance between the two dimers of uricase homotetramer and is thus disadvantageous to enzyme activity. With a larger side chain than glycine, glutamic acid at position 83 increases the steric hindrance at the cornering between the two α-helixes of uricase monomer and thus adversely affects the accurate positioning of active residues of the enzyme. Based on these predictions, site-directed mutagenesis was performed to obtain chimera H_1-2_P_3_H_4_P_5-6_H_7-8_ (E24D & E83G), which was more homologous to dHU and more active than PBC.

## 4. Materials and Methods

### 4.1. Microorganisms, Vectors, and Materials

Host strain *E. coli* BL 21 Star^TM^ (DE3) and vector pET-22b(+) were from Invitrogen (Carlsbad, CA, USA). DNA polymerase, DNA marker, T4 DNA ligase, and restriction endonucleases *Nde* I and *Hind* III were from Fermentas (Waltham, MA, USA). A polymerase chain reaction (PCR) amplification kit (including PCR buffer and dNTP mix) was obtained from Takara (Shiga, Japan). The plasmid mini kit I and PCR product recovery kit were purchased from Omega Bio-Tek (Norcross, GA, USA). Uric acid standard was from Sigma-Aldrich (St. Louis, MO, USA). All other reagents were of analytical grade.

### 4.2. Construction of dHU, wPU, and Porcine-Human Chimeras

A codon-optimized full length dHU gene with two premature mutations at codon 33 and 187 replaced by CGA (arginine) was designed and synthesized based on the hypothetic protein sequence of HU. Restriction sites *Nde* I and *Hind* III were introduced at the 5′-and 3′-terminus respectively. After digestion and ligation, *dhu* was inserted into the multiple cloning site of pET-22b(+) vector. The wPU gene was also chemically synthesized in a similar manner and constructed into a pET-22b(+) vector. A PBC gene was accidentally obtained in our previous study on DNA shuffling between *wpu* and *dhu*.

In the exon replacement study, each exon in *wpu* was replaced by the corresponding exon in *dhu* to investigate the combined effect of all mutations accumulated during evolution in one exon on the activity of uricase. The plasmid and strains in this study are shown in Table 2. The procedures are briefly described by taking exon 3 as an example. The nucleotide sequence of exon 3 in *dhu* was amplified using primers 15 and 16 in Table S1. In order to substitute the corresponding exon in *wpu*, two bulky fragments containing exons 1–2 and exons 4–8 were amplified from *wpu* using primers 1 & 2 and 5 & 12. With overlapping sequences, the two bulky fragments were spliced, with exon 3 amplified from *dhu* by overlap extension PCR (SOE-PCR). Replacement of other exons was carried out in a similar manner. In the subsequent exon restoration study, the three most deleterious exons in *dhu* were replaced by the corresponding exons in *wpu* one after another to gradually restore the activity of dHU. The primers used for splicing were designed according to the nucleotide sequence of each exon in *dhu* and *wpu* by Primer Premier 5.0 and are shown in Table S1. All the chimeric genes were confirmed by DNA sequencing. All the recombinant plasmids were transformed into *E. coli* BL 21. Positive clones were screened on an LB plate supplemented with 100 μg/mL ampicillin.

### 4.3. Expression and Purification of Porcine–Human Chimeras (PHC), wPU, and PBC

For expression of each chimeric enzyme, a seed culture was prepared by overnight cultivation in an LB medium containing 100 μg/mL ampicillin and then inoculated into a fresh fermentation medium. After cultivation at 37 °C for 4 h, protein expression was induced by the addition of isopropyl-β-d-thiogalactoside (IPTG; 0.2 mM final concentration), followed by further cultivation for 6 h. Cells were harvested by centrifugation at 10,000× *g* for 10 min at 4 °C. The cells’ pellets were re-suspended in a lysis buffer and homogenized by sonication. The cell lysate was centrifuged at 10,000× *g* for 20 min at 4 °C to completely remove the cell debris. Solid ammonium sulfate was added to the recovered supernatant to 10% saturation at 4 °C. The precipitate was re-dissolved in an Na_2_CO_3_–NaHCO_3_ buffer (0.1 M, pH 10.3), loaded onto an anion exchanger (Q-Sepharose Fast Flow), and eluted using 0.5 M NaCl. Fractions showing uricolytic activities were pooled and loaded onto a Sephacryl S-300 column (GE Healthcare, Chicago, IL, USA). After elution with an Na_2_CO_3_–NaHCO_3_ buffer (0.1 M, pH 10.3), target fractions were collected and stored at 4 °C for further analysis.

### 4.4. Protein Analysis and Enzymatic Assay

SDS-PAGE was carried out to determine the homogeneity of purification and the molecular mass of the chimeras [33]. Protein content was measured by the Bradford method with bovine serum albumin as a standard [34]. The enzymatic activity of purified uricase was determined spectrophotometrically by monitoring the decrease of uric acid in absorbance at 293 nm as described previously [35]. Solutions of uric acid were prepared in 50 mM sodium borate buffer pH 8.5 to a final concentration of 100 μM. Purified uricase was dissolved in the same buffer and mixed with the substrate. The enzymatic reaction was carried out at 37 °C for 3 min with monitoring of absorbance at 293 nm every 4 s. The maximum rate of decrease in the absorbance per minute was calculated. An extinction coefficient of 12,300 M^−1^·cm^−1^ for uric acid was used [36]. One unit (U) of enzymatic activity was defined as the amount of enzyme that consumes 1 μmol of uric acid per minute. Specific activity was expressed as U/mg. The kinetic parameters *K_m_* and *V_max_* of dHU, wPU, H_1-2_P_3_H_4_P_5-6_H_7-8_ (E24D & E83G), and PBC were estimated by the double reciprocal plot method. Using different concentrations of uric acid (0.001–0.144 mM), the enzyme activity was assayed as described above. The turnover number (*k_cat_*) was calculated based on the value of *V_max_*, the concentration of the purified enzyme, and the molecular weight. *K_m_* was calculated by the Lineweaver–Burk plotting.

### 4.5. Multiple Protein Sequence Alignment and Homology Modeling

Amino acid sequences of three functional mammal uricases (pig, baboon, and dog) and three functional chimeric uricases (canine–human, porcine–human, and pig–baboon) were retrieved from GenBank or obtained from related patents. Multiple protein sequence alignment of those functional Uox with H_1-2_P_3_H_4_P_5-6_H_7-8_ and dHU was generated using Clustal X version 2.0 (Conway Institute, UCD, Dublin, Ireland) to analyze the inconsistent residues across these sequences.

In order to predict the influence of specific amino acid residues (E24 and E83) on the activity of uricase, the 3D structure of mammalian uricase deposited by Ortlund and colleagues in the PDB database (PDI ID: 4MB8) was used as a template to model the 3D structures of mutated chimeric uricases using MOE 2010.10 (Chemical Computing Group Inc., Montreal, QC, Canada) [30].

### 4.6. Site-Directed Mutagenesis of E24D and E83G

Site-directed mutagenesis was implemented to obtain E24D and E24D & E83G mutants of H_1-2_P_3_H_4_P_5-6_H_7-8_. Two pairs of primers (P13 & 26 and P24 & 25) with introduction of mutation E24D were designed against the gene fragments coding for amino acid residues 1–36 and 22–304 of H_1-2_P_3_H_4_P_5-6_H_7-8_ (see Table S1) using Primer Premier 5. The two gene fragments were amplified and spliced by SOE-PCR, resulting in mutant H_1-2_P_3_H_4_P_5-6_H_7-8_ (E24D). Mutation E83G was further introduced into the E24D mutant by similar procedures, using primer pairs P27 & 24 and P13 & 28. After transformation into *E. coli* BL 21, positive clones harboring the recombinant plasmid were obtained and mutations were confirmed by DNA sequencing. Chimeric enzymes were expressed and purified according to the same procedures described above.

### 4.7. Simulation of Interaction between Uricase H_1-2_P_3_H_4_P_5-6_H_7-8_ (E24D & E83G) and Uric Acid

In order to predict the interaction between uricase H_1-2_P_3_H_4_P_5-6_H_7-8_ (E24D & E83G) and uric acid, structural modeling of uric acid to the binding site of uricase H_1-2_P_3_H_4_P_5-6_H_7-8_ (E24D & E83G) was performed *in silico* by employing MOE 2010.10. 4MB8 was superposed to *Arthrobacter globiformis* uricase (PDI ID: 2YZB), whose crystal structure was complexed with uric acid. With three conserved residues in the active site (Q236, R187, and F170), a conserved water molecule involved in catalytic activity and the two residues (N262 and T68) hydrogen-bonded to it restrained, the 4MB8 structure complexed with uric acid was modeled and further used as a template to model the 3D structures of uricase H_1-2_P_3_H_4_P_5-6_H_7-8_ (E24D & E83G). Interaction between uric acid and the binding site of uricase H_1-2_P_3_H_4_P_5-6_H_7-8_ (E24D & E83G) was simulated in 2D view and 3D view by employing MOE 2010.10 and Visualizer module of Discovery studio (DS) 3.0 package (Accelrys Software, Inc., San Diego, CA, USA), respectively.

### 4.8. Effect of pH and Temperature on the Activity of H_1-2_P_3_H_4_P_5-6_H_7-8_ (E24D & E83G)

The effect of pH and temperature on the enzyme activity of H_1-2_P_3_H_4_P_5-6_H_7-8_ (E24D & E83G) was investigated by determining its uricolytic activity at a pH range of 6.0–11.0 using different buffer systems (NaH_2_PO_3_–Na_2_HPO_3_ 6.0–7.0, borate buffer pH 7.0–9.0, Na_2_CO_3_–NaHCO_3_ buffer pH 9.0–11.0) as well as a temperature range of 20–70 °C. The results were expressed as the percentage of activity obtained at either the optimum pH or the optimum temperature. wPU and PBC were also included for comparison. pH stability of H_1-2_P_3_H_4_P_5-6_H_7-8_ (E24D & E83G) was measured by pre-incubating (in a ratio of 1:1) the enzyme solution in the abovementioned buffer systems for 30 min at room temperature and subsequently measuring its activity at 35 °C. The percentage of residual enzyme activity was calculated by assuming enzyme activity at the beginning of the reaction as 100%. Thermal stability of the enzyme was determined by pre-incubating the enzyme at different temperatures for 30 min. The percentage of residual enzyme activity was determined after cooling or warming the sample to 35 °C.

## 5. Conclusions

In conclusion, we applied a semi-rational approach to develop a chimeric uricase for hyperuricemia and gout. Exon replacement and exon restoration were designed to identify the more detrimental exons and restore enzyme activity. Sequence alignment and homology modeling were successfully applied to predict favorable mutations. With increased homology with dHU, higher activity than PBC, and good pH and thermal stability, the resulting chimera H_1-2_P_3_H_4_P_5-6_H_7-8_ (E24D & E83G) is a more promising drug candidate for hyperuricemia and gout.

## Figures and Tables

**Table 1:** Kinetic parameters for Uox of dHU, wPU, H_1-2_P_3_H_4_P_5-6_H_7-8_ (E24D & E83G), and PBC.

Uricase	*K_m_* (Μm)	*k_cat_/*(s)	*k_cat_/K_m_/*(s/μM)	Specific Activity	Homology with dHU (%)
dHU	ND	ND	ND	ND	100
wPU	10.12 ± 0.10	23.54 ± 0.03	2.33	4.32 ± 0.07	87.5
H_1-2_P_3_H_4_P_5-6_H_7-8_ (E24D & E83G)	5.37 ± 0.09	30.95 ± 0.01	5.76	6.03 ± 0.06	91.45
PBC	7.51 ± 0.04	27.24 ± 0.03	3.62	5.19 ± 0.08	89.5

Values are means ± standard deviation of five replicates; ND, not determined.

**Table 2:** List of plasmids and strains in this study.

Strain/Plasmid	Description	Source/Reference
Strain		
*E.coli* BL 21 Star^TM^ (DE3)	Cloning host	Invitrogen
Plasmid		
pET-22b(+)	T7 promoter, Amp^R^	EMD Biosciences
pET-22b(+)_H_1-2_	coding for H_1-2_P_3-8_	This study
pET-22b(+)_H_3_	coding for P_1-2_H_3_P_4-8_	This study
pET-22b(+)_H_4_	coding for P_1-3_H_4_P_5-8_	This study
pET-22b(+)_H_5_	coding for P_1-4_H_5_P_6-8_	This study
pET-22b(+)_H_6_	coding for P_1-5_H_6_P_7-8_	This study
pET-22b(+)_H_7-8_	coding for P_1-6_H_7-8_	This study
pET-22b(+)_P_6_	coding for H_1-5_P_6_H_7-8_	This study
pET-22b(+)_P_5-6_	coding for H_1-4_P_5-6_H_7-8_	This study
pET-22b(+)_P_3-5-6_	coding for H_1-2_P_3_H_4_P_5-6_H_7-8_	This study
pET-22b(+)_24	coding for H_1-2_P_3_H_4_P_5-6_H_7-8_(E24D)	This study
pET-22b(+)_24–83	coding for H_1-2_P_3_H_4_P_5-6_H_7-8_(E24D & E83G)	This study

## Supplementary Materials

Supplementary materials can be found at http://www.mdpi.com/1422-0067/17/5/764/s1.
