# Supplementary material for: Development of Therapeutic Chimeric Uricase by Exon Replacement/Restoration and Site-Directed Mutagenesis
Source: Int J Mol Sci. 2016 May 20;17(5):764. doi: 10.3390/ijms17050764 (PMC4881585; doi:10.3390/ijms17050764)
Supplement: Supplementary file 1 [file ijms-17-00764-s001.pdf]

Supplementary Materials: Development of Therapeutic Chimeric Uricase by Exon Replacement/Restoration and Site-Directed Mutagenesis

Guangrong Xie, Weizhen Yang, Jing Chen, Miaomiao Li, Nan Jiang, Baixue Zhao, Si Chen, Min Wang and Jianhua Chen

Table S1. Primers used for exon replacement and restoration (overlapping sequences designed for SOE-PCR are colored).

Primer	Description	Sequence
P1	Forward primer for *wpu* exon1 with restriction site *Nde* I (underlined)	5′-GGAATTCCATATGGCTCATTACCGTAATGACT-3′
P2	Reverse primer for *wpu* exon 2	5′-GCCTTTGAACTTCGCCAGGACATTAACTGTG-3′
P3	Forward primer for *wpu* exon 3	5′-ATCAAAAGCATAGAAACTTTTGCTGTGAC-3′
P4	Reverse primer for *wpu* exon 3	5′-CTTTTCAAAACGCTTCCAAGGAACTTCTTCCAC-3′
P5	Forward primer for *wpu* exon 4	5′-AATGGAGTTAAGCATGTCCATGCATTTA-3′
P6	Reverse primer for *wpu* exon 4	5′-ATTCCTTATCTGTTCAACCTCACAGAAGTGCG-3′
P7	Forward primer for *wpu* exon 5	5′-GGACCTCCAGTCATTCATTCTGGAATC-3′
P8	Reverse primer for *wpu* exon 5	5′-CCAGGTGGCCTCAAAGTCCACATCTCTGCCC-3′
P9	Forward primer for *wpu* exon 6	5′-GACACTGTTAGGAGCATTGTCCTGCAGA-3′
P10	Reverse primer for *wpu* exon 6	5′-CTCAGGAACCTGGCCCAGGGTGAGCACCTGGATGT-3′
P11	Forward primer for *wpu* exon 7	5′-ATAGAAGATATGGAAATCAGCCTGCCAA-3′
P12	Reverse primer for *wpu* exon 8 with restriction site *Hind* III (underlined)	5′-CCCAAGCTTTCACAGCCTTGAAGTCAGC-3′
P13	Forward primer for *dhu* exon1 with restriction site *Nde* I (underlined)	5′-GGAATTCCATATGGCCCACTACCATAACAAC-3′
P14	Reverse primer for *dhu* exon 2	5′-AAAAGTTTCTATGCTTTTGATTTCTTTAAACTTTGCCA-3′
P15	Forward primer for *dhu* exon 3	5′-TGTCCTGGCGAAGTTCAAAGGCATCAAAAGCATAGAAG-3′
P16	Reverse primer for *dhu* exon 3	5′-GCATGGACATGCTTAACTCCATTCTTTCCAAGATGCTT-3′
P17	Forward primer for *dhu* exon 4	5′-GAAGCGTTTTGAAAAGAATGGAGTTAAGCATGTC-3′
P18	Reverse primer for *dhu* exon 4	5′-GAATGAATGACTGGAGGTCCACTTCTCAGCTGTTCAA-3′
P19	Forward primer for *dhu* exon 5	5′-TGAACAGATAAGGAATGGACCCCAAGTCATTC-3′
P20	Reverse primer for *dhu* exon 5	5′-ACAATGCTCCTAACAGTGTCCCAGGTAGCCTTGAAGT-3′
P21	Forward primer for *dhu* exon 6	5′-ACTTTGAGGCCACCTGGGACACCATTCGGGAC-3′
P22	Reverse primer for *dhu* exon 6	5′-GCTGATTTCCATATCTTCTATCGCAGGAACTCGGCTC-3′
P23	Forward primer for *dhu* exon 7	5′-TGGGCCAGGTTCCTGAGATAGAAGATATGGAAATC-3′
P24	Reverse primer for *dhu* exon 8 with restriction site *Hind* III (underlined)	5′-CCCAAGCTTTCACAGTCTTGAAGACAACTTCC-3′
P25	Forward primer for amino acid fragment 22–304 of $H_{1-2}P_3H_4P_{5-6}H_{7-8}$ and should be paired with P24	5′-GAAGGATATGGTAAAAGTTTTGCATATTCAGCGAGATGGAAAGT-3′
P26	Reverse primer for amino acid fragment 1–36 of $H_{1-2}P_3H_4P_{5-6}H_{7-8}$ and should be paired with P13	5′-ACTTTCCATCTCGCTGAATATGCAAAACTTTTACCATATCCTTC-3′
P27	Forward primer for amino acid fragment 79–304 of $H_{1-2}P_3H_4P_{5-6}H_{7-8}$ (E24D) and should be paired with P24	5′-GGCAAAGTTTAAAGGCATCAAAAGCATAGAAG-3′
P28	Reverse primer for amino acid fragment 1–84 of $H_{1-2}P_3H_4P_{5-6}H_{7-8}$ (E24D) and should be paired with P13	5′-GATGCCTTTAAACTTTGCCAAGACATGAAC-3′

The primer in red is the overlapping sequence used in SOE-PCR.
